# Ischemic Stroke Revealing Libman-Sacks Endocarditis as the Initial Manifestation of Lupus Erythematosus With Secondary Antiphospholipid Syndrome: A Case Report

**DOI:** 10.7759/cureus.70870

**Published:** 2024-10-05

**Authors:** Georgio El Koubayati, Nicolas Sandakly, Ingrid Jabbour, Fady Haddad, Majd Khalil

**Affiliations:** 1 Internal Medicine and Clinical Immunology, Lebanese University Faculty of Medicine, Beirut, LBN; 2 Internal Medicine, Lebanese University Faculty of Medicine, Beirut, LBN; 3 Internal Medicine and Clinical Immunology, Lebanese Hospital Geitaoui - University Medical Center, Beirut, LBN; 4 Cardiology, Lebanese Hospital Geitaoui - University Medical Center, Beirut, LBN

**Keywords:** antiphospholipid antibody syndrome (aps), blood culture negative endocarditis, libman sacks endocarditis (lse), neuropsychiatric sle, sytemic lupus erythematosus

## Abstract

Libman-Sacks endocarditis (LSE), also known as nonbacterial thrombotic endocarditis (NBTE), is a rare condition characterized by noninfectious lesions on the heart valves with the deposition of thrombi. NBTE is most commonly linked to advanced malignancy, systemic lupus erythematosus, and antiphospholipid syndrome (APS). In many cases, NBTE is only diagnosed postmortem during autopsies, with embolization being the most frequent clinical manifestation. A high level of clinical suspicion is essential for diagnosis. Despite treatment involving anticoagulation and addressing the underlying etiology, the prognosis remains generally poor. We present the case of a 40-year-old woman admitted with new-onset dysarthria and upper motor weakness. Clinical evaluation, laboratory testing, and imaging revealed a stroke accompanied by valvular vegetations, which were linked to secondary APS. The patient was diagnosed with LSE and treated with anticoagulation. Neurological manifestations, such as embolization, are common in NBTE, often occurring in otherwise asymptomatic patients. Despite management of the acute condition, NBTE continues to be associated with high morbidity and mortality.

## Introduction

Libman-Sacks endocarditis (LSE) was first described in 1924 in four patients with atypical, sterile verrucous lesions affecting the valvular and mural endocardium [1]. These lesions, pathologically distinct from other forms of endocarditis, were identified as a feature of systemic lupus erythematosus (SLE) [2,3]. Early autopsy studies revealed that 35-65% of lupus patients had Libman-Sacks vegetations, although these were often clinically silent with minimal hemodynamic impact [3,4]. Later autopsy series reported a lower prevalence and smaller vegetations [5,6]. However, echocardiography later demonstrated that thickened, functionally impaired cardiac valves, prone to hemodynamic deterioration, were common in SLE patients [7]. The association between LSE and antiphospholipid antibodies (aPLs) was first noted in 1985 in a young woman with SLE and lupus anticoagulants (LAs) [8]. Similar cases were subsequently reported in four other SLE patients and one patient with primary antiphospholipid syndrome (APS) [9,10]. By 1989, research indicated that aPLs contributed to valvular heart disease in SLE, leading to the understanding that these valve lesions are part of APS [7,11].

We present the case of a young woman diagnosed with LSE complicated by an ischemic stroke, which was the initial manifestation of lupus erythematosus with secondary APS.

## Case presentation

**Figure 1:**
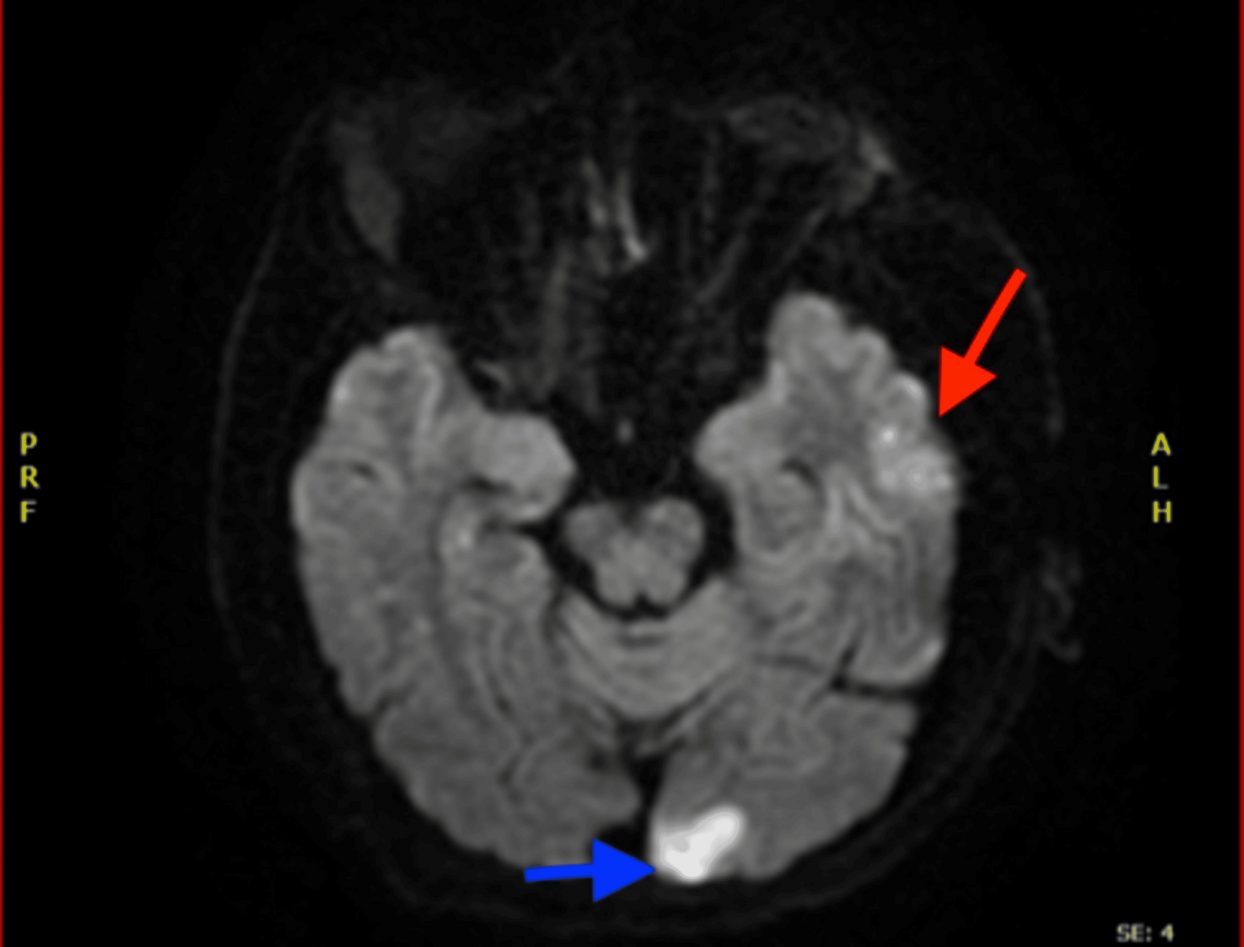
MRI of the brain showing T2 FLAIR high signal intensities in the left occipital lobe (blue arrow) and left parietal lobe (red arrow), suggestive of showering emboli

A 40-year-old woman presented to the Lebanese Hospital Geitaoui University Medical Center with dysarthria and upper motor weakness. The patient had a history of multiple spontaneous abortions. An initial physical examination was unremarkable except for mild dysarthria and unilateral left upper limb motor weakness with preserved sensation. The last known normal was determined to be 10 hours before admission. She had no history of arterial or venous limb thrombosis, ischemic heart disease, or pulmonary thromboembolism. The patient denied experiencing joint tenderness or swelling, photosensitivity, skin lesions, or ulcers. A 12-lead ECG showed a normal sinus rhythm with no abnormal findings. Initial laboratory studies revealed normocytic normochromic anemia, severe thrombocytopenia, and elevated CRP. During the initial examination, the mild dysarthria and unilateral upper motor weakness progressed into a significant neurological deficit, raising suspicion of an ongoing cerebrovascular accident (CVA). An urgent cerebral CT scan was performed without intravenous contrast, revealing left-sided parietal and occipital signs of ischemia. A subsequent magnetic resonance angiography showed multiple foci of restriction on diffusion-weighted sequences, suggesting showering emboli (Figure 1).

**Figure 2:**
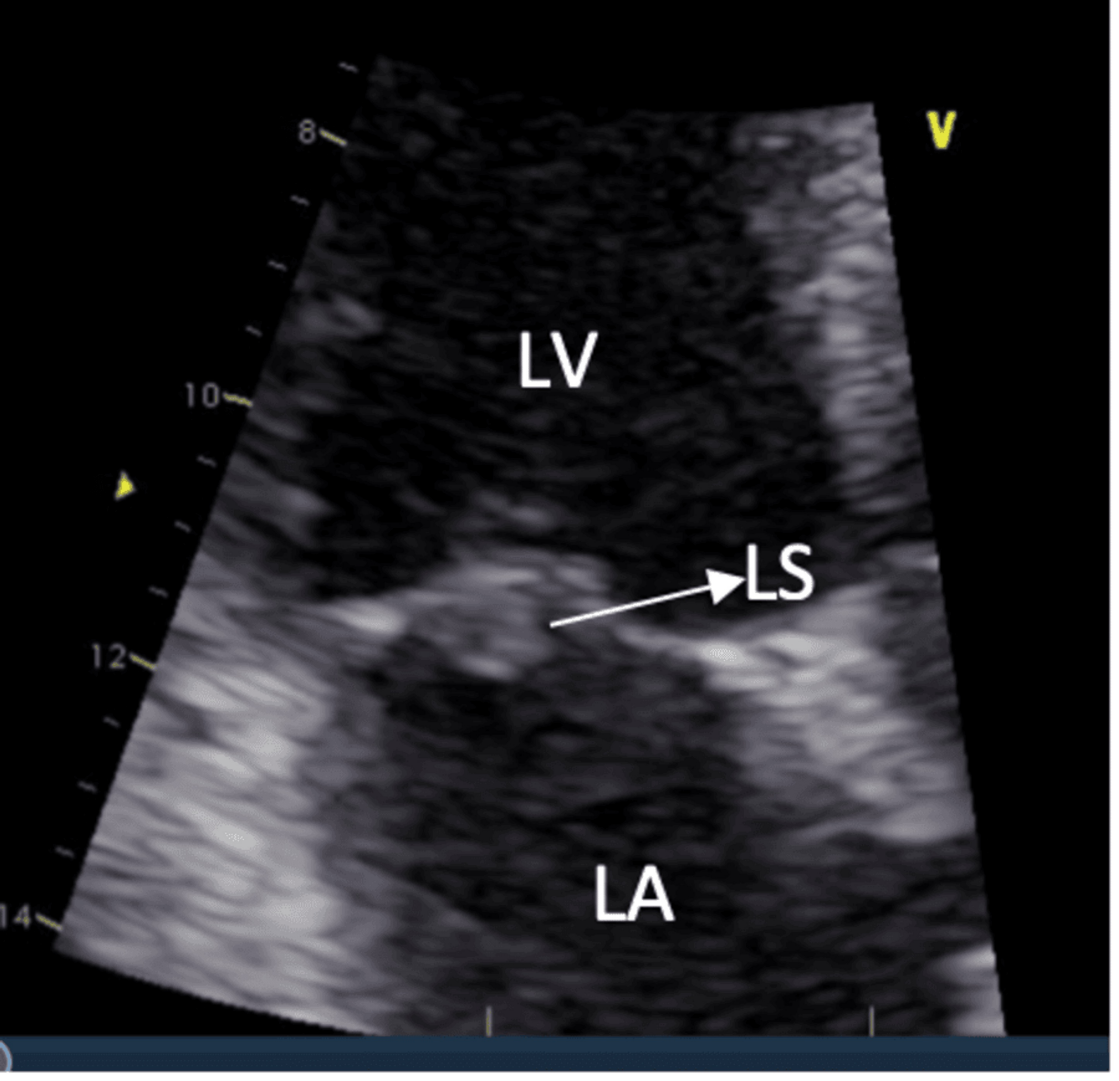
TTE showing an oscillating mass on the posterior leaflet of the mitral valve LA, left atrium; LV, left ventricle; LS, Libman-Sacks endocarditis; TTE, transthoracic echocardiogram

The patient was admitted to the neurology stroke service for further workup. On the second day of admission, she experienced worsening dysarthria and muscle weakness. A transthoracic echocardiogram (TTE) revealed an incidental finding of a 7 mm mobile mass on the atrial side of the posterior mitral leaflet, suggestive of endocarditis (Figure 2).

**Figure 3:**
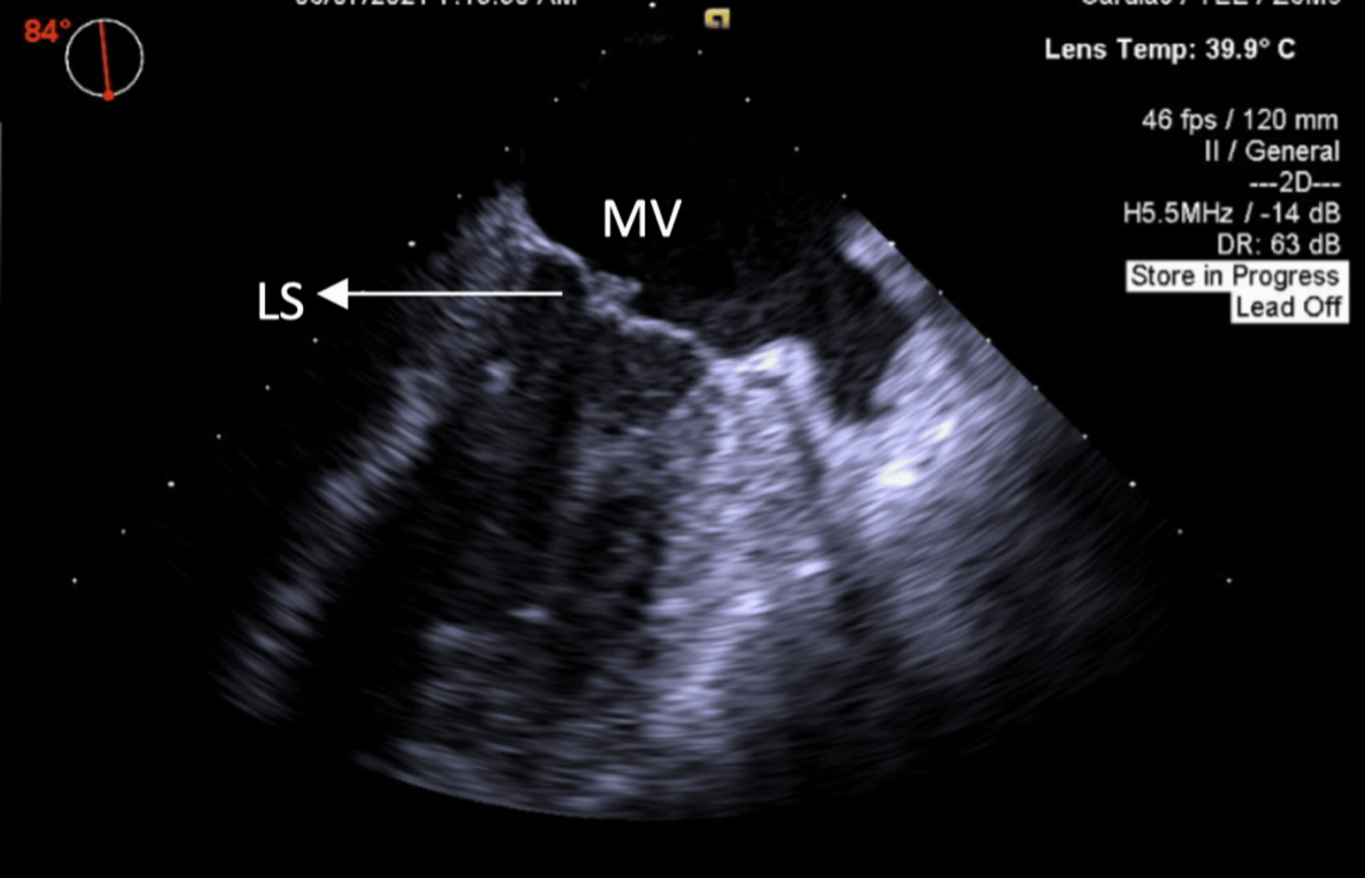
TEE confirming LS on the atrial side of the posterior mitral leaflet LS, Libman-Sacks endocarditis; MV, mitral valve; TEE, transesophageal echocardiogram

A transesophageal echocardiogram further corroborated this finding (Figure 3).

Cardiac contractility was normal, with an ejection fraction of 68% and no valvulopathy. Blood cultures were collected, and a total of three sets remained negative after seven days of incubation. Direct antiglobulin testing was positive, along with a positive hemolytic workup (Table 1).

**Table 1:** Initial laboratory results demonstrating anemia, thrombocytopenia, and a positive hemolytic workup

Laboratory exam	Results	Reference range
Hemoglobin (g/dl)	9.3	12-16
Platelets (cells/mm^3^)	11,000	130,000-400,000
Reticulocyte count (%)	11	0.5-1.5
Partial thromboplastin time (s)	32	32
Total bilirubin (mg)	1.9	0-1.3
Direct bilirubin (mg)	0.9	0-0.3
Creatinine (mg)	0.78	0.4-1
Haptoglobin (g/L)	<0.1	0.3-2
Coombs Direct	Positive	-
CRP (mg/dl)	71	0-6
Procalcitonin (ng/mL)	0.01	-

An immunological origin was subsequently suspected, and further investigation revealed a positive antinuclear antibody (ANA), positive anti-double-stranded DNA, low complement levels, and elevated aPL titers (Table 2).

**Table 2:** Autoimmune workup confirming active SLE and APS ANA, antinuclear antibody; anti-dsDNA, anti-double-stranded DNA; APS, antiphospholipid syndrome; SLE, systemic lupus erythematosus; SSA, SS-A native; SSB, SS-B native

Investigation	Results	Reference range
Serum immunoglobulin		
IgA (g/L)	1.24	0.8-3
IgM (g/L)	1.01	0.4-2.5
IgG (g/L)	15.78	6-16
ANA	1/320	Not available
SSA	Negative	Not available
SSB	Negative	Not available
Anti-dsDNA IgG (IU/mL)	50.5	0-29.9
Anti-Sm (U/mL)	3.2	0-7
ANCA-C (AU/mL)	2.8	≤19
ANCA-P (kU/L)	1.2	<1.4
C3 (g/L)	0.7	0.9-1.8
C4 (g/L)	0.1	0.1-0.4
Anti-B2 glycoprotein IgM (SMU/mL)	4.8	≤20
Anti-cardiolipin IgG (GPL U/mL)	27.5	≤15
Anti-cardiolipin IgM (MPL U/mL)	1.1	<12.5
Anti-cardiolipin IgA (APL U/mL)	2	≤11
Anti-phospholipid IgG (U/mL)	>100	<14
Anti-phospholipid IgM (U/mL)	1.2	<14
Lupus anticoagulant	Positive	Not available
Protein C (%)	112	70-140
Protein S (%)	106	59-118

**Figure 4:**
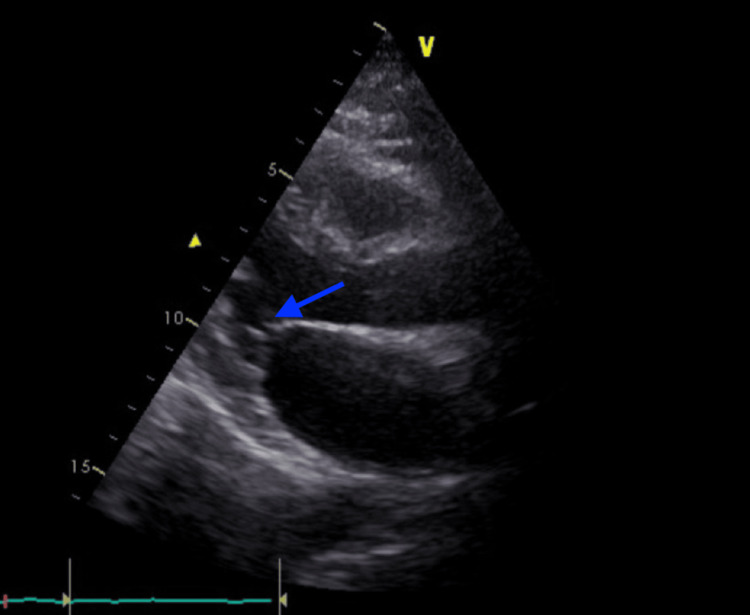
TTE performed four weeks after treatment initiation showing complete resolution of the endocarditis The disappearance of vegetations on the mitral valve is indicated (blue arrow). TTE, transthoracic echocardiogram

Based on these findings, a diagnosis of non-bacterial thrombotic endocarditis was made. Further investigation included EDTA whole blood PCR to detect mutations associated with thrombophilia, which revealed no heterozygous mutations. However, two genes showed homozygous mutations: MTRR 66 and MTHFR 1298. The patient was started on corticosteroids (prednisone 1 mg/kg daily). The thrombocytopenia rapidly improved, and the patient’s neurological deficits partially subsided. She began a prolonged course of corticosteroids and was therapeutically anticoagulated with warfarin. Physiotherapy was initiated concurrently to aid recovery from the partial disability caused by the ischemic event. The patient was discharged home on warfarin 5 mg with an INR goal of 2.5-3, prednisolone 25 mg daily, and hydroxychloroquine 400 mg daily. A repeat TTE performed four weeks after treatment initiation showed complete resolution of the endocarditis (Figure 4).

## Discussion

LSE is considered a rare entity due to its asymptomatic or mild clinical presentation, with a prevalence ranging from 0.9% to 1.6%, often discovered postmortem. Notably, postmortem studies have reported a prevalence of 30-50% in patients with SLE [12]. However, with the advent of echocardiography as a diagnostic tool and the increased survival of lupus patients, LSE has been more frequently reported in the literature over the past decade. The left-sided heart valves are predominantly affected. A study by Moyssakis et al. evaluating the prevalence and progression of LSE in SLE patients found LSE in 38 out of 342 patients; among these, 24 had mitral involvement, 13 had aortic involvement, and only one had tricuspid involvement [13].

Differentiating LSE from infective endocarditis can be challenging. In our case, the patient initially presented with neurological manifestations, hemolytic anemia, and thrombocytopenia. Initial and repeated blood cultures remained negative after seven days of incubation, and serological tests for common pathogens associated with infective endocarditis were also negative. The diagnosis of nonbacterial thrombotic endocarditis (NBTE) was further supported by the presence of positive aPLs, a history of recurrent spontaneous abortions, and the patient’s clinical improvement following the initiation of corticosteroids and anticoagulation therapy.

The treatment of NBTE-associated CVAs primarily involves anticoagulation [14-16] and addressing the underlying etiology. Anticoagulation should be initiated only in the absence of brain hemorrhage. In some cases, the presence of persistent vegetations on heart valves with recurrent ischemic CVA necessitates surgical intervention [15]. It is important to note that, in contrast to infectious endocarditis, surgical intervention in NBTE allows for the preservation of valve tissue, although it does not completely protect against relapse. A similar approach may be applicable if NBTE is related to a resectable tumor. However, in the context of SLE and APS, treatment focuses on managing the underlying disease. While corticosteroids aim to reduce inflammation, they can lead to tissue scarring and fibrosis, which may predispose patients to subsequent valvular damage [17,18].

## Conclusions

We diagnosed a case of LSE in a 40-year-old female patient who presented with neurological manifestations and ongoing coagulopathies. This case highlights several important aspects of LSE, particularly as an initial presentation of SLE secondary to APS, manifesting as multiple cerebral ischemic strokes.

This case emphasizes the necessity of maintaining a high index of suspicion for LSE in the differential diagnosis of unexplained CVAs. Although confirming the diagnosis can be challenging, an appropriate interpretation of the clinical, biological, and immunological profiles of the patient is essential for initiating adequate treatment, preventing further complications, and improving patient outcomes.
